# ﻿*Cyphocallipus africanus* sp. nov.: first native callipodid millipede from continental Africa (Diplopoda, Callipodida)

**DOI:** 10.3897/zookeys.1253.161612

**Published:** 2025-09-26

**Authors:** Pavel Stoev, Nesrine Akkari

**Affiliations:** 1 National Museum of Natural History at the Bulgarian Academy of Sciences, Tsar Osvoboditel Blvd. 1, 1000 Sofia, Bulgaria National Museum of Natural History at the Bulgarian Academy of Sciences Sofia Bulgaria; 2 Pensoft Publishers, Prof. G. Zlatarski Str. 12, Sofia, Bulgaria Pensoft Publishers Sofia Bulgaria; 3 Naturhistorisches Museum Wien, Burgring 7, 1010 Wien, Austria Naturhistorisches Museum Wien Wien Austria

**Keywords:** Cave, Ibero-African distributions, Morocco, Myriapoda

## Abstract

The millipede *Cyphocallipus africanus***sp. nov.** (Diplopoda, Callipodida) from Morocco is described, proving the occurrence of native species of the order Callipodida on the African continent. The only male specimen of the new species was discovered in the cave Maala Abladir near the village of Bab Taza in Rif Mountain Range in northern Morocco. The species differs morphologically from *C. excavatus* Verhoeff, 1909, known from southern Spain, by the specific shapes of the coxosternal processes, the protective lamella of the distal part of the telopodite, and the sternal lamella. In the light of the new finding, the opportunity is taken to revise the taxonomic position of the Iberian genera of Callipodida, hitherto grouped in the subfamily Cyphocallipodinae Verhoeff, 1909. The opinion expressed by other authors—that the Iberian genera are separate from the Balkan–Anatolian genus *Dorypetalum* Verhoeff, 1900—is confirmed, and a new status is proposed for the Iberian taxa as a separate family, Cyphocallipodidae**stat. nov.** The genus *Cyphocallipus* represents another example of a millipede genus with an Ibero-African distribution, together with genera such as *Origmatogona* Ribaut 1913, *Ceratosphys* Ribaut, 1920 (Chordeumatida), and *Boreviulisoma* Brolemann, 1928 (Polydesmida).

## ﻿Introduction

Twelve orders of millipedes are hitherto reported from the African continent: Polyxenida Verhoeff, 1934; Sphaerotheriida Brandt, 1833; Glomerida Leach, 1814; Siphonophorida Newport, 1844; Polyzoniida Gervais, 1844 (only in South Africa and Madagascar); Polydesmida Leach, 1815; Chordeumatida Pocock, 1894; Stemmiulida Pocock, 1894; Julida Brandt, 1833; Spirostreptida Brandt, 1833; Siphonocryptida Pocock, 1894; and Spirobolida Bollman, 1893. Among the recorded orders, the Glomerida and Julida are absent in the Afrotropical region, having a Palearctic distribution (Enghoff 2015; Enghoff et al. 2015), whereas Siphonocryptida is known from a single species, *Hirudicryptus canariensis* (Loksa, 1967) from the Canary and the Madeiran archipelagos (Enghoff 2010). No native species of the order Callipodida Pocock, 1894 have been described from Africa to date, although mislabelled (and erroneous) records, or such based on female specimens of uncertain taxonomic assignment, were reported by various authors from North Africa (see below).

The order Callipodida is a moderately abundant group of diplopods, which currently includes more than 150 described species (catalogued by Stoev et al. 2008; see also Enghoff et al. 2015). In recent years, several new extant species have been described from the USA (Shelley and Richart 2014), China (Liu and Tian 2015; Akkari et al. 2023; Chen et al. 2023), Spain (Gilgado et al. 2020), and Vietnam (Nguyen et al. 2023, 2025), as well as a new fossil family from the Cretaceous amber of Burma (Stoev et al. 2019). The order has a fragmented global distribution (Shear et al. 2003; Stoev et al. 2008; Shelley and Golovatch 2011; Enghoff et al. 2015), with major centres of species diversity in regions such as the Mediterranean Europe, particularly on the three main peninsulas—the Pyrenees, Apennines, and Balkans—and their neighbouring islands (families Callipodidae Bollman, 1893, Schizopetalidae Vergoeff, 1909, Dorypetalidae Verhoeff, 1900). In Asia, the order exhibits a patchy distribution that spans from the westernmost parts in Türkiye, Syria, Palestine, and Israel (families Schizopetalidae and Dorypetalidae), through the central regions in Iran, Afghanistan, Pakistan, Tajikistan, Turkmenistan, and Kyrgyzstan (families Schizopetalidae, Caspiopetalidae Lohmander, 1931), to Southeast Asia, including southern and central China, Thailand, Laos, and Vietnam (families Caspiopetalidae, Sinocallipodidae Zhang, 1993 and Paracortinidae Wang & Zhang, 1993). The order is also represented in North America in two disjunct areas—the southwestern United States and northwestern Mexico, occupied by Tynommatidae Hoffman, 1980, and a large region in the eastern and central United States that extends southward into Coahuila and Nuevo León, Mexico, inhabited by both Tynommatidae and Abacionidae Shelley, 1979.

The first to record the order Callipodida from Africa was Attems (1927), who described *Brölemannia africana* Attems, 1927 from Senaar (currently Sennar) in Sudan and reported *Brölemannia asiaeminoris* Verhoeff, 1898 [as *B. byzantina asiae minoris* (sic!)] from the same locality. Both taxa are currently assigned to the genus *Eurygyrus* C.L. Koch, 1847 in the family Schizopetalidae (Hoffman and Lohmander 1964; Stoev et al. 2008; Vagalinski et al. 2014). However, Stagl and Stoev (2005) studied the type material of *B. africana* housed in the Naturhistorisches Museum Wien (NHMW) and pointed out that these records are erroneous, most probably due to the mislabelling of the material. Both taxa were collected by the Austrian naturalist Theodor Kotschy, who undertook several expeditions to Türkiye, the Near East, Egypt, and Sudan between 1836 and 1839. It is very likely that he (or somebody else after him) mixed up the jars resulting in the labels being interchanged. Apart from the fact that the genus *Eurygyrus* is restricted to an area from the Peloponnese in the west through Türkiye to the western part of Levant in the east, another argument in support of the claim that the material was mislabelled is in the museum’s acquisition book, where the entry corresponding to the specimen collection lists “Taurus” (Toros Mountains in Türkiye). According to Stagl and Stoev (2005), the same may have happened to the material of *Eurygyrus asiaeminoris*, a species that also occurs in Taurus Mountains and adjacent areas in Türkiye, which was recently recorded from Cyprus (Vagalinski et al. 2014).

Brоlemann (1931) recorded a female callipodidan (without generic assignment) from the gardens of the University of Algiers in Algeria. A few years later, Manfredi (1939) recorded another female specimen of an uncertain taxonomic position from Uadi Derna [Wadi Derna] in Libya. She concluded that, given the extreme scarcity of specimens, it cannot be ruled out that these records belong to a species introduced from Europe, which had not yet become abundant in their new habitat.

Compared to the fauna of Algeria and especially Tunisia, from where many new species of Diplopoda have been described in recent decades (e.g. Abrous-Kherbouche and Mauriès 1996; Read 2005; Akkari and Enghoff 2008; Golovatch et al. 2009; Akkari et al. 2013), the diplopods of Morocco remain poorly studied. The latest significant contribution was by Schubart (1960). Later, some species (mostly from caves) were described by Mauriès (1985) and Enghoff and Reboleira (2019). Akkari et al. (2009) reviewed all species of the order Julida in North Africa and reported 20 species from Morocco. Akkari and Mauriès (2018) compiled a similar checklist of Polydesmida species occurring in North Africa.

This article describes the first representative of the order Callipodida found in Africa. Additionally, we take the opportunity to revise the status of the geographically well-defined subfamily Cyphocallipodinae Verhoeff, 1909 (Dorypetalidae).

## ﻿Material and methods

The material (one male, holotype) has been obtained for study from Mr Per Djursvoll, Department of Natural History of the University Museum of Bergen, Norway, and is currently housed in the Myriapoda collection of the Naturhistorisches Museum Wien (**NHMW**). It was collected by a team of speleologists from the Grupo de Exploraciones Subterráneas of Priego (Spain) in a cave in Morocco in 2005. The specimen was studied and photographed in NHMW using a Nikon DS-Ri2 camera mounted on a Nikon SMZ25 stereomicroscope, using NIS-Elements Microscope Imaging Software with an Extended Depth of Focus (EDF). Scanning electron micrographs (SEM) were obtained of the gonopods of *C. excavatus* using a JEOL JSM-6335F scanning electron microscope in the Natural History Museum of Denmark (NHMD). All images were edited in Adobe Photoshop CS2024 and assembled in Adobe InDesign CS2024.

We follow the terminology of the different parts of the gonopods of *Cyphocallipus* Verhoeff, 1909, as established by Mauriès (1978). Setal arrangement follows Hoffman and Lohmander (1964).

## ﻿Results

### ﻿Taxonomy

The Iberian genera of Callipodida have hitherto been placed in a separate subfamily Cyphocallipodinae of the family Dorypetalidae. According to Verhoeff (1909), the following characters unite the Balkan–Anatolian genus *Dorypetalum* Verhoeff, 1900 with the Spanish–Portuguese taxa: 1) sternum of the gonopods almost completely separated at the median line into two paramedian halves, each partly fused with the coxa; 2) each half is extended into a strong process; 3) gonopod telopodite thin, long, curved in an arch shape and always accompanied by a horn-like flagellum (Verhoeff 1909; Hoffman and Lohmander 1964). At present the family comprises two subfamilies: Dorypetalinae with one genus and seven species (Stoev and Enghoff 2006; Stoev et al. 2008; Enghoff et al. 2015) and Cyphocallipodinae with three genera and five species, the fifth of which is added in the present study (Spelda 2015; Gilgado et al. 2020). Hoffman (2009: 644) stated that the inclusion of the subfamily Cyphocallipodinae in the same family as Dorypetalinae “cannot be justified” but he deferred from elevating the subfamily to a family rank awaiting a comparative study of the gonopod musculature in the group. Hoffman’s (2009) statement came at a time when only the morphology of the genus *Cyphocallipus* was relatively well known, thanks to Mauriès’ (1978) redescription of the species. In recent years, new information has been accumulated about the external anatomy of the other two genera in Cyphocallipodinae: *Lusitanipus* Mauriès, 1978 and *Dorycallipus* Verhoeff, 1909. This includes the redescription of *Lusitanipus alternans* (Verhoeff, 1893) and the description of a second species, *L. xanin* Gilgado, 2020, from Spain (Reboleira and Enghoff 2015; Gilgado et al. 2020).

With all these data available, we believe that the grouping of Dorypetalinae and Cyphocallipodinae, two morphologically and geographically distinct lineages, is artificial, and we propose their separation in two distinct families of order Callipodida. The new families are diagnosed as follows:

#### ﻿Cyphocallipodidae Verhoeff, 1909, stat. nov.

All setae in posterior position on pleurotergites from PT6 onwards; male pre-gonopodal legs 4–7: tarsae with claws and without significant modifications on coxae and prefemora, apart from the prefemora being slightly enlarged. Gonopods: very complex, with several (2, 3 or more) large and complexly arranged coxosternal processes, and a long and arcuate telopodite accompanied by an accessory pseudoflagellum; distal part of telopodite with a protective lamella. Female second legs significantly reduced and modified.

**Type genus.***Cyphocallipus* Verhoeff, 1909.

**Distribution.** Iberian Peninsula; Rif Mountains (Morocco).

#### ﻿Dorypetalidae Verhoeff, 1900

Seta *a* migrates in posterior position on PT8 or remains in anterior position on all PTs; male pregonopodal legs 4–7: tarsae of without claws; coxae and prefemora with various modifications (outgrowths). Gonopods: much simpler, coxosternal processes less elaborated, telopodite and additional process (called prefemoral but similar to pseudoflagellum) well separated from each other; distal part of telopodite simple, never with a protective lamella. Female second legs normal.

**Type genus.***Dorypetalum* Verhoeff, 1900.

**Distribution.** Balkan Peninsula; Romania, Hungary, and Türkiye.

#### ﻿Family Cyphocallipodidae stat. nov., char. emend.

**Description.** Small to moderately large callipodidans, adults from approx. 20 mm (*Dorycallipus*) to approx. 56–60 mm (*Cyphocallipus*). Number of pleurotergites: 44–62. Forehead of males either concave (*Cyphocallipus*, *Dorycallipus*) or unmodified and convex (*Lusitanipus*). A pair of small knobs present on epicranium in the currently known species of *Cyphocallipus*. Pleurotergites either with primary crests only (*Dorycallipus*, *Cyphocallipus*, *Lusitanipus xanin* Gilgado, 2020) or with primary and secondary crests (*Lusitanipus alternans*). All ozopores situated between the crests. Pleurotergal setae usually in anterior position on PTs 1–4 (occasionally 1 or 2 setae could be posterior on collum), some setae migrate posteriorly on PT5; all setae in posterior position from PT6 onwards. Coxal sacs present on leg-pairs 3–22 in *C. excavatus* and on leg-pairs 3–16 in *Lusitanipus xanin*. Male pre-gonopodal legs 3–7: tarsae with claws, prefemora usually slightly enlarged. Gonopods: sternum divided, with sternal lamella protecting the base of gonocoxa; coxosternum with 2 or 3 large processes; telopodites parallel to each other; long and arcuate, attached at the posterior end of coxa, curving anteriad and then pointing posteriad, in situ protruding well beyond the edge of gonocoel and approaching leg-pair 9; telopodite with an accessory process called pseudoflagellum (or Hornflagellum in German literature), fused with the base of telopodite; pseudoflagellum either is very thin and curved, following the curvature of the telopodite (*Cyphocallipus* and *Dorycallipus*) or straight and rod-like (*Lusitanipus*), not adjacent to the telopodite; basal and proximal part of telopodite without any processes, distal part of telopodite divided at the end into a canal branch and a protective lamella that extends beyond it.

**Females**: posterior lateral edge of second metazonum either produced caudal as a large subtriangular lobe (*C. excavatus* Verhoeff, 1909; unknown in *C. africanus* sp. nov.) or margin describing subtle tentative insinuation of process in that position (*L. xanin*) or normal (*L. alternans*).

#### ﻿Included taxa

##### Genus *Cyphocallipus* Verhoeff, 1909

**Type species.***Cyphocallipus excavatus* Verhoeff, 1909, by monotypy.

*C. excavatus* Verhoeff, 1909. SPAIN. Type locality. Algeciras. The species range extends approximately 500 km, from Gibraltar in the west to Almería in the east, and includes the Spanish provinces of Cádiz, Málaga, Granada, and Almería (Spelda 2015). According to Jiménez-Valverde et al. (2015), previous records from Alicante (Hoffman 2009; Ortuño et al. 2013) belong to an undescribed species of *Cyphocallipus* and not to *C. excavatus*. It occurs beneath stones and within the leaf litter of maritime *Pinus* woodlands and maquis and has so far been found at elevations up to 1300 m (Kime and Enghoff 2011).

*C. africanus* sp. nov. MOROCCO: Rif Mountains, cave (present study).

##### Genus *Dorycallipus* Verhoeff, 1909

**Type species.***Dorycallipus arcuum* Verhoeff, 1909, by monotypy.

*D. arcuum* Verhoeff, 1893 SPAIN: exact type locality unknown; the material was sent to Verhoeff from southern Spain without details of location (Kime and Enghoff 2011). The genus *Dorycallipus* is currently under review based on new material from Spain (Akkari et al. in prep.).

##### Genus *Lusitanipus* Mauriès, 1978

**Type species.***Lusitanipus alternans* (Verhoeff, 1893), by monotypy.

*L. alternans* (Verhoeff, 1893) PORTUGAL: in caves, mesovoid shallow substrate and surface habitats from Outil-Cantanhede massif throughout the Sicó-Condeixa and Alvaiázere karst chains, down to Abrigo de Tomar I Cave in Ourém; the species was also reported from Buraco da Moura Cave in Serra da Estrela massif (Reboleira and Eusébio 2023). Recent records under stones in Leiria District suggest it is abundant in surface habitats (Gilgado 2024).

*L. xanin* Gilgado, 2020 SPAIN: León province, known from a small cave near its entrance, and under stones (Gilgado et al. 2020).

In terms of distribution, the three genera are well-separated geographically, except probably for the province of Alicante, where *Dorycallipus* and *Cyphocallipus* ranges may overlap. The genus *Lusitanipus* is well separated in the west and north-west of the Iberian Peninsula and is distant from *Dorycallipus*, which is confined to the eastern part. *Cyphocallipus* occupies a large area in the south of the peninsula, with one species occurring in North Africa.

###### ﻿Genus Cyphocallipus

Taxon classificationAnimaliaCallipodidaDorypetalidae

Verhoeff, 1909

6F65A348-40DC-5D23-87D2-B44C5D06C9FD

####### Diagnosis.

Body with 53–59 PTs. Forehead of male concave; a pair of small knobs present on epicranium of both known species of *Cyphocallipus*; pleurotergites: with longitudinal crests, which are spaced apart in their posterior part; metazona not much elevated compared to prozona (in *Lusitanipus* and *Dorycallipus* posterior part are elevated); Collum: with longitudinal furrows and indistinct setae in the posterior half and at the side flaps; Coxal sacs: from 3^rd^ to 22^nd^ pair of legs. Gonopods: with a large sternal lamella and three coxosternal processes, two of them much longer than the 3^rd^ between them, the foremost with an excavation for the telopodite; telopodite long and thin pseudoflagellum following its curvature. Female (unknown in *C. africanus*): posterior lateral edge of second metazonum produced caudal as a large subtriangular lobe over lower side of third. Second leg-pair greatly reduced (Verhoeff 1909; Hoffman 2009; Spelda 2015).

####### Comment.

In his review of the members of the order Callipodida from the Iberian Peninsula, Spelda (2015) provided a comprehensive bibliographic catalogue of the records of the genus *Cyphocallipus* and *C. excavatus*, along with photographs of key taxonomic characteristics of the species. Therefore, we do not include a bibliographic review here.

###### ﻿ Cyphocallipus africanus sp. nov.

Taxon classificationAnimaliaCallipodidaDorypetalidae

34B24800-D548-59F1-BB4D-94F7A303214D

https://zoobank.org/3423D8F0-EB21-4D2F-A0EE-CB34914C0C50

Figs 1, 2

####### Diagnosis.

A species of the genus *Cyphocallipus*, differing from *C. excavatus* in having a semicircular rounded protective lamella at the distal part of telopodite (vs subrectangular in *C. excavatus*), bearing smaller fold at the margin than in *C. excavatus*; coxosternal process *m* with apically rounded and not distally expanded process *k*, and a much shorter process *j* (1/3 of process *k* vs 1/2 of process *k* in *C. excavatus*); coxosternal process *i* with a straight distal part; coxosternal process *i*’ broad, incised distally, posterior part of distal incision oval, posterior proximal margin triangularly oval; sternal lamella similar to *C. excavatus* in position but lower. Table 1 provides a comparison of the main taxonomic characters between *Cyphocallipus africanus* sp. nov. and *C. excavatus*.

**Table 1.** Main taxonomic characters of gonopods that distinguish *Cyphocallipus africanus* sp. nov. from *C. excavatus*.

Character	*C. excavatus*	*C. africanus* sp. nov.
Sternal lamella: posterior to telopodite and coxal processes	Large, broad (Fig. 6E)	Less developed, lower (Fig. 1E)
Broad, incised distally posterior coxosternal process *i*’	Posterior part of distal incision triangular; posterior proximal margin triangular (Figs 7D, 8F)	Posterior part of distal incision oval, posterior proximal margin oval (Fig. 2D)
Coxosternal process *m* Forked to processes *j* and *k*	Process *j* twice shorter than process *k*; process *k* is expanded distally and somewhat triangular (Figs 7B, 8E, 9A)	Process *j* is 1/3 of length of process *k*; *k* apically rounded and not distally expanded (Figs 1F, 2B)
Coxosternal process *i*	Distal part curved (Figs 6F, 8E)	Distal part straight (Figs 1E, 2A)
Distal part of telopodite: enlarged, with a rounded buckle at the base of the protective lamella	Protective lamella subrectangular, with a fold at posterior margin (Figs 7B, D, E, 9A)	Protective lamella more rounded at margin, semicircular, with a very small fold at margin (Fig. 2C, D)

####### Etymology.

The specific epithet emphasizes the discovery of the first native member of the order Callipodida on the African continent. Adjective.

####### Material examined.

• ***Holotype***: adult male, Morocco, Tanger-Tetouan-Al Hoceima region, Chefchaouen Province, Bab Taza, cave Maala Abladir, 35°04'07.3"N, 5°06'19.6"W, 14.VIII.2005, leg. G.E.S. [Grupo de Exploraciones Subterráneas of Priego], NHMW MY10657.

####### Description.

**Figure 1.**
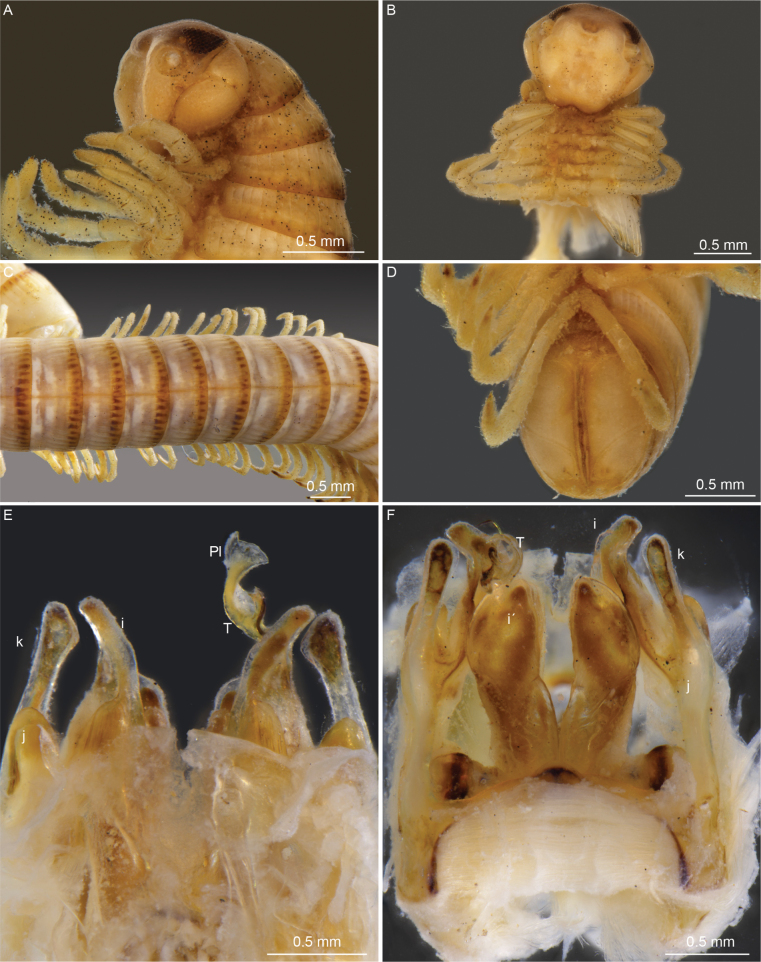
*Cyphocallipus africanus* sp. nov., holotype. A. Head and anteriormost pleurotergites, lateral view; B. Head, frontal view; C. Pleurotergites, dorsal view; D. Telson, ventral view; E. Gonopods, posterior view; F. Gonopods, anterior view. Abbreviations: coxosternal processes k, j I, i´, T – telopodite, Pl – protective lamella.

Body cylindrical, length *circa* 56 mm, maximal body diameter *circa* 2.85 mm. 58 PTs + Telson. Colour in life unknown; colour of preserved specimen: head frons and vertex yellow alveolate. Male head concave with the characteristic for the genus pair of knobs on epicranium (Fig. 1A, B); Eyes: 53 ommatidia in 10 rows (Fig. 1A). Organ of Tömösváry twice as large as largest ommatidium. All pleurotergites yellow, with light-brown transverse band on posterior margin (Fig. 1C). Antennae broken, brownish, ratio: 2>3>5>4>6>7 (vs 5>2>4>6>3>1>7). Legs yellow; claws darker. Pleurotergal crests touching each other at anterior part of pleurotergite (prozonum) and becoming separate and thinning towards pleurotergite end (Fig. 1C); 5+5 pleurotergal crests between ozopores on midbody pleurotergites. Ozopores situated between crests 5 and 6, very small and difficult to spot.

***Legs***: Leg-pairs 1 and 2 reduced and more setose than the rest, with postfemoral and tibial brushes and tarsal combs. Leg-pairs 3–5 with postfemoral and tibial brushes; legs 1–5 (also postgonopodal legs) with prefemur, femur, postfemur and tibia micropapillate; leg-pair 2 with posterior gonopore, prefemora of legs 3–7 slightly enlarged and covered with dense setae. Coxal sacs large and conspicuous at least until leg-pair 12.

***Telson* (Fig. 1D)**: anal valves: with smaller dorsal and larger ventral plates; dorsal plates with one microseta each; hypoproct tripartite, median sclerite largest, subtrapezoidal, bearing a pair of macrosetae situated in the middle. Lateral sclerites smaller, subtriangular, with one seta each. Epiproct: with two moderately long spinnerets.

**Figure 2.**
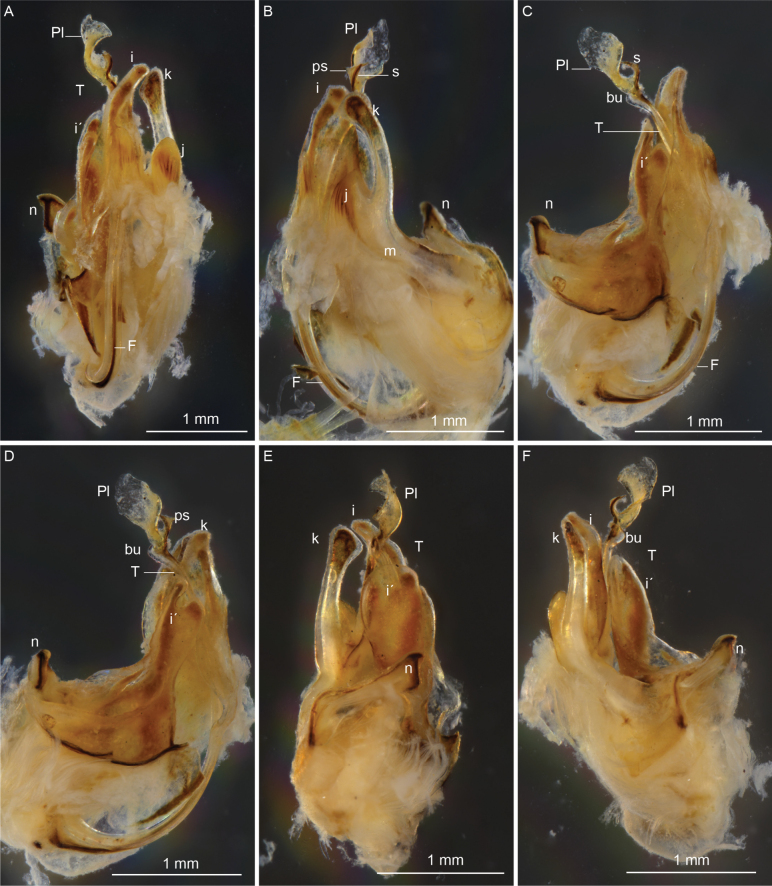
*Cyphocallipus africanus* sp. nov., holotype, gonopods. A. Anterior view; B. Lateral view; C, D. Mesal view; E, F. Posterior view. Abbreviations: coxosternal processes n, k, j I, i´, T – telopodite, F – pseudoflagellum, bu – buckle, Pl – protective lamella, S – solenomere, ps – parasolenomere.

***Gonopods* (Figs 1E, F, 2A–F)**: gonopod comprising a long and arcuate telopodite (T), attached at the inner posterior end of gonocoxa and pointing posterioventrad, accompanied by a flagelliform accessory process (pseudoflagellum - F), following the curvature of telopodite. Distal part of telopodite enlarged, with a rounded buckle (*bu*) at the base of the protective lamella (*Pl*). Protective lamella covering partially the solenomere (s) and parasolenomere (*ps*); lobe more rounded at margin, semicircular, with a very small fold at the margin. Sternal lamella large, broad, posterior to the telopodite and the coxal processes, less developed and lower compared to *C. excavatus*; Coxosternum with three large processes *i*’, *i*, and *m*. Posterior coxosternal process *i*’ broad, incised distally, posterior part of distal incision oval, posterior proximal margin triangularly oval; coxosternal process *m* forked into processes *j* and *k*; process *j* is 1/3 of the length of process *k*; *k* apically rounded and not distally expanded as in *C. excavatus*. Coxosternal process *i* straight.

**Female.** Unknown.

####### Distribution.

**Figure 3.**
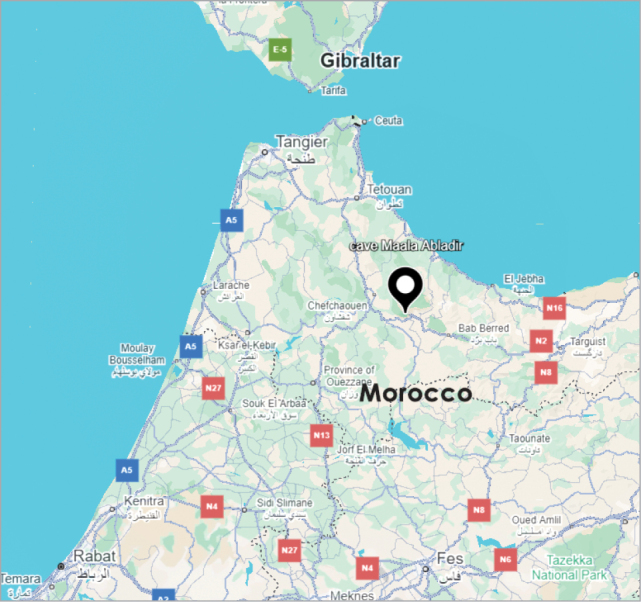
The location of the cave Maala Abladir in Morocco. Source: Google Earth Pro (2025), v. 7.3. Google LLC. Retrieved August 26, 2025, from https://www.google.com/earth/.

**Figure 4.**
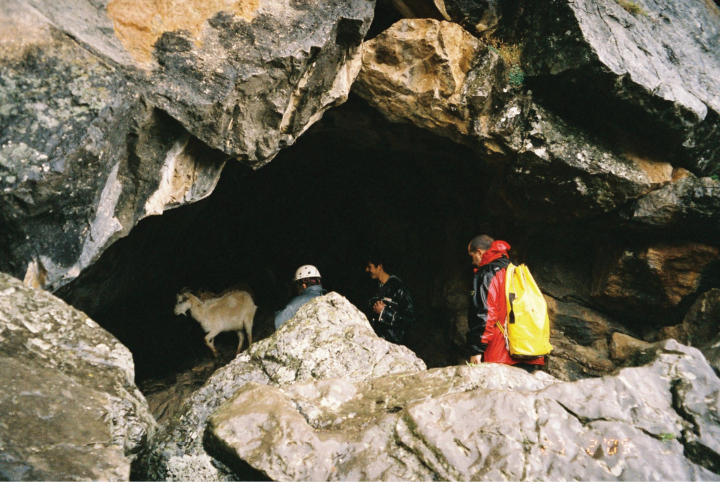
The entrance of cave Maala Abladir. Photo: Miguel Ángel Maestre.

**Figure 5.**
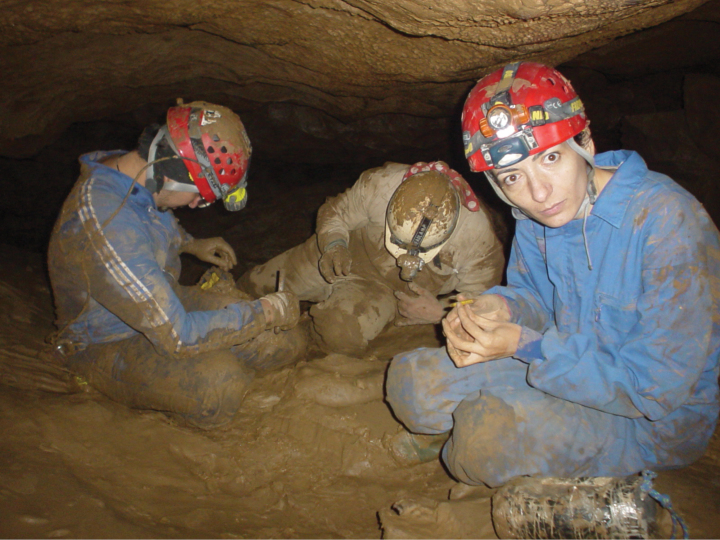
Speleologists from the Grupo de Exploraciones Subterráneas of Priego, Spain, exploring the cave. Photo: Miguel Ángel Maestre.

Known only from the type locality in Morocco. Bab Taza is a small town in Talassemtane National Park in the Rif Mountains (Moutaouakil pers. comm.) (Fig. 3). The cave Maala Abladir has relatively large entrance (Fig. 4) and two floors. Clay covers much of the area, and the cave ends with a pool (Fig. 5). In a paper summarizing the results of studies on hypogean beetles of the family Cholevidae in Morocco, Fresneda and Fadrique (2006) reported the *Speonemadus maroccanus* (Jeannel, 1936) from the Maala Abladir. To our knowledge, this is the only other animal hitherto recorded from the cave.

####### Habitat.

Although collected from a cave, the new species does not exhibit any traits of troglomorphism, leading us to presume that it was only seeking shelter there.

####### Comments.

The specimen is heavily infested by fungi (Fig. 1A, B). Most likely they are representatives of the ectoparasitic fungi of the order Laboulbeniales Engler, 1898, as described in other members of Callipodida from the Iberian Peninsula (Reboleira and Enghoff 2015).

###### ﻿ Cyphocallipus excavatus

Taxon classificationAnimaliaCallipodidaDorypetalidae

Verhoeff, 1909

581D550E-3BFE-5315-B3A1-C85A221AA2BF

Figs 6, 7, 8, 9

####### Material examined.

• 1 male, Spain, Almería, Sierra de Gádor, Canchal Antenas II, MSS, Félix, 36°52'49.53"N, 2°45'29.32"W, 1300 m, 11.XII.2018, Centro de Investigación de Colecciones Científicas de la Universidad de Almería, Cecoual-Dpto. Biol. Geol. col. Pablo Barranco leg. (Figs 3A–D, 4A–E); • 1 male, 3 females, Spain, Málaga Province, Cómpeta, Casa de la Mina, Pine Forest, 15.I.2006, leg. D. Kime, det. J-P. Mauriès (National Museum of Natural Hostory, Sofia – Myriapoda collection) (Fig. 3E, F); • 1 male, Spain, Órgiva, 7.X.1977, Briganti, Parodi, Zoia leg., ex. Mus. Verona, G. Osella ded. 1983 (Scanning electron micrographs).

####### Description.

**Figure 6.**
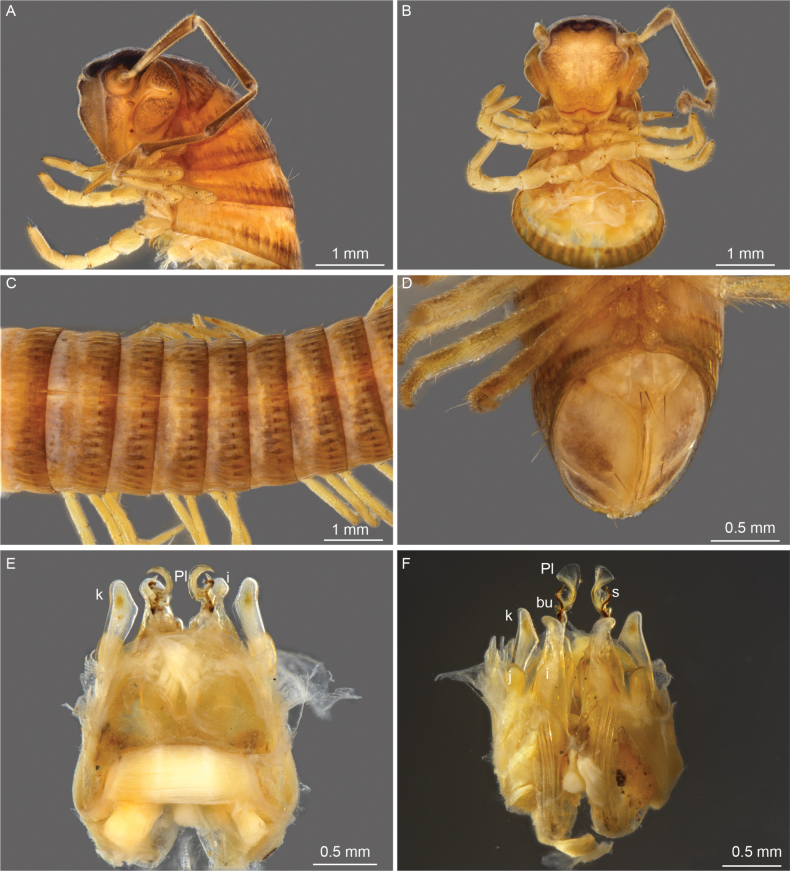
*Cyphocallipus excavatus*, males. A–D. From Almeria; E, F. From Cómpeta; A. Head and anteriormost PTs, lateral view; B. Head, frontal view; C. Pleurotergites, dorsal view; D. Telson, ventrolateral view; E. Gonopods, posterior view; F. Gonopods, anterior view. Abbreviations: coxosternal processes k, j I, T – telopodite, bu – buckle, Pl – protective lamella, s – solenomere.

**Figure 7.**
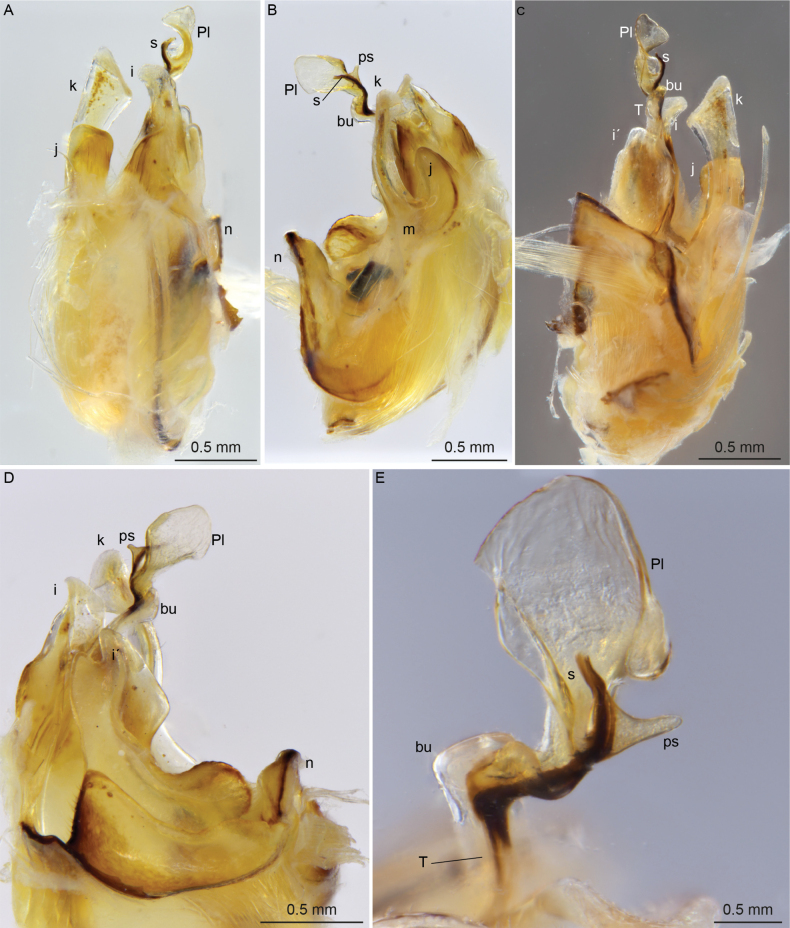
*Cyphocallipus excavatus*, male from Almeria, gonopods; A. Posterior view; B. Lateral view; C. Anterior view; D. Mesal view; E. Closeup of distal part of telopodite. Abbreviations: n, k, j I, i´, T – telopodite, bu – buckle, Pl – protective lamella, S – solenomere, ps – parasolenomere.

**Figure 8.**
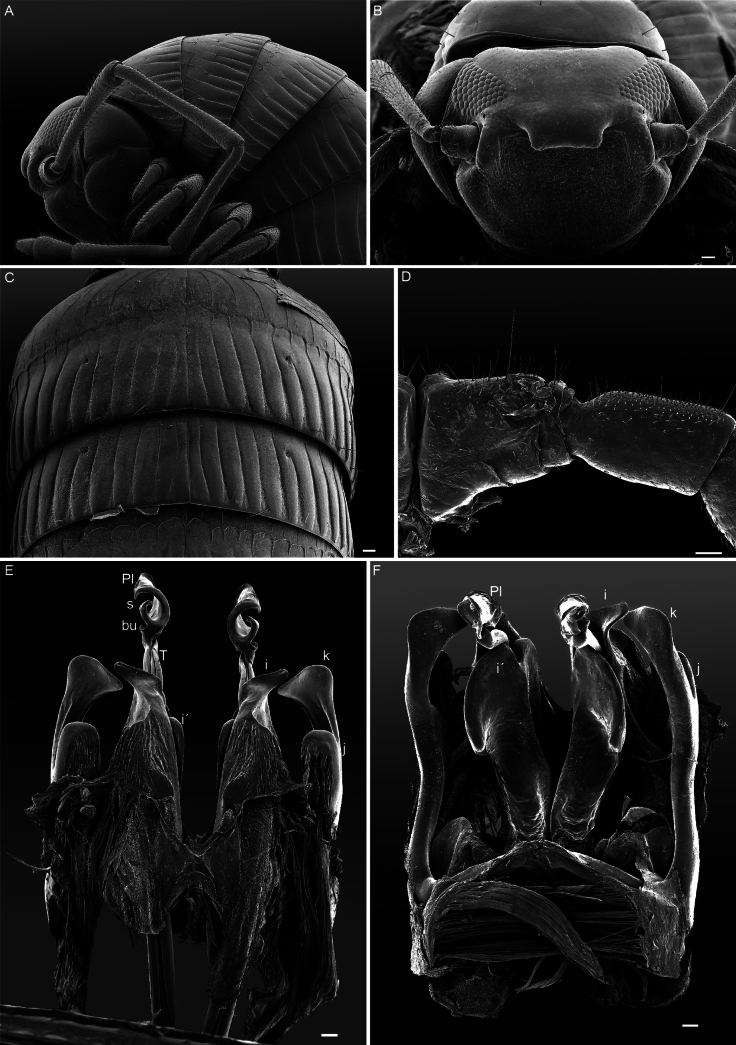
*Cyphocallipus excavatus*, male from Órgiva, SEM. A. Head and anteriormost pleurotergites, lateral view; B. Head, dorsal view; C. Midbody pleurotergites, dorsal view; D. Leg 7, anterior view; E. Gonopods, anterior view; F. Gonopods, posterior view. Abbreviations: coxosternal processes k, j I, i´, F – pseudoflagellum, T – telopodite, bu – buckle, Pl – protective lamella, S – solenomere. Scale bars: 0.1 mm.

**Figure 9.**
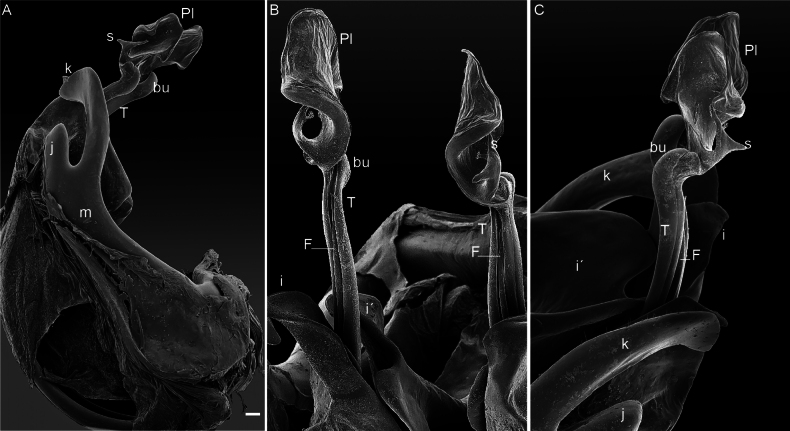
*Cyphocallipus excavatus*, male from Órgiva, SEM, gonopods. A. Lateral view; B, C. Close up of distal part of telopodites. Abbreviations: m, k, j I, i´, T – telopodite, F – pseudoflagellum, bu – buckle, Pl – protective lamella, S – solenomere, ps – parasolenomere. Scale bar: 0.1 mm (A).

This species has already been redescribed by Mauriès (1978), Hoffman (2009), and Spelda (2015). However, here, we present habitus images of the species and document the species gonopod morphology, using both light micrography and scanning electron microscope imagery. Thus, we provide more accurate and detailed information on key taxonomic features (Figs 6–9). Table 1 summarizes the main distinguishing characteristics of the two species.

## ﻿Discussion

*Cyphocallipus excavatus* is widely distributed in southern Spain and Gibraltar and was recently mapped by Kime and Enghoff (2011) and Spelda (2015: map 3). The new species occurs only approximately 120 km from the nearest known record of *C. excavatus* across the Strait of Gibraltar. According to Spelda (2015: 8) “The species (*C. excavatus*) seems to be more common in coastal biotopes or in stream valleys, although it also occurs higher in the mountains up to 1300 m. While juveniles can be found at various sites, adult males are usually found in cool, moist places, such as springs on northern slopes”.

Zoogeographically, Morocco is particularly interesting, as it is the only North African country that hosts millipedes of the southern/Gondwanan taxon, specifically Spirostreptidea, which are known to inhabit the Atlas Mountains and the coastal areas (Shelley and Golovatch 2011). According to Shelley and Golovatch 2011: 55), “Spirostreptidea’s occupation of Morocco therefore antedates the xerification of the Saharan region that partitioned the Moroccan population from the rest of the suborder”. Additionally, the mostly tropical tribe Eviulisomatini Brölemann 1916 (Polydesmida, Paradoxosomatidae Daday, 1889) is known in the Palaearctic region with three species of *Jeekelosoma* Mauriès, 1985 from caves in Morocco, and genus *Boreviulisoma* Brolemann, 1928 with three species from Spain, Portugal, and Morocco (Mauriès 1985; Reboleira and Enghoff 2013; Enghoff and Reboleira 2019).

Shelley and Golovatch (2011) speculated that the absence of Callipodida in North Africa (despite being well represented in the Iberian Peninsula and some Mediterranean islands), and respectively of Spirostreptidea in Europe, is due to the fact that Callipodida and Spirostreptidea spread into Iberia and North Africa, respectively, after Laurasia and Gondwana had split, and neither could extend into the other continent. With the recent discovery of *C. africanus* sp. nov. in Morocco, this hypothesis may need reconsideration, particularly regarding Callipodida, which exhibits a distribution pattern similar to that of Chordeumatida and Glomerida. Тhe genus *Cyphocallipus* may have arisen in the late Miocene between the Messinian salinity crisis, 5.96 million years ago, and the opening of the Strait of Gibraltar (Zanclaean Flood) about 5.33 million years ago, somewhere in the region of the Iberian Peninsula and northwest Africa. The ancestors of *Cyphocallipus* may have dispersed widely in the region during the partial drying of the Mediterranean Sea. Before the Messinian Crisis, the spread of organisms from North Africa to the Iberian Peninsula was not possible due to the presence of two water corridors connecting the Atlantic with the Mediterranean Sea: the Betian Corridor through southern Spain and the Riphian Corridor through northern Morocco, the latter remaining open until the Messinian salinity (Krijgsman 2002; Sanmartín 2003).

Though less abundant and diverse, the Callipodida might even have a wider distribution in North Africa, extending to Libya in the east as suggested by the discoveries of female individuals of uncertain taxonomic position in Algeria and Libya (Brolemann 1931; Manfredi 1939), which might also be of native origin.

Many plants and animals show Iberian–North African distribution patterns, which are often restricted only to the Baetic and Rif mountains on both sides of the Gibraltar Strait. Among Diplopoda, similar ranges are demonstrated by the following taxa (see also Reboleira and Enghoff 2013):

Genus *Origmatogona* Ribaut 1913 (Chordeumatida, family Chamaesomatidae Verhoeff, 1913): comprises six species (and one doubtful subspecies) from Morocco, France, and Spain. Interestingly, *Origmatogona strinatii* Manfredi, 1956 is known from the cave of Friouato in Taza Province, while the remaining five congeners are distributed in caves and subterranean habitats in France and Spain (Manfredi 1956; Mauriès 1990, 2014).

Genus *Ceratosphys* Ribaut, 1920 (Chordeumatida, family Opisthocheiridae Ribaut, 1913). The genus comprises 25 species from South Spain, France, the Canary and Balearic islands, and Morocco (Kime and Enghoff 2021). Likewise, *Origmatogona strinatii* and *C. maroccana* Mauriès, 1985 occurs in the Friouato cave in Morocco (Mauriès 1985).

Genus *Boreviulisoma* Brolemann, 1928 (Polydesmida, family Paradoxosomatidae, tribe Eviulisomatini Brölemann 1916) is an example of a taxon with a North African–Iberian distribution. Three species are known from Spain, Portugal (cave), and Morocco (Boulhaut S of Rabat, Volubilis N of Meknés. and Tasloumt near Tahanaout, 35 km S of Marrakech) (Reboleira and Enghoff 2013).

Genus *Archipolydesmus* Attems, 1898 (Polydesmida, Polydesmidae). This genus comprises 13 species currently distributed in Spain, France, Morocco, and Algeria. *Archipolydesmus altibaeticus* Gilgado & Enghoff, 2015 is morphologically very similar to *A. maroccanus* Attems, 1898 (Gilgado et al. 2015). According to Gilgado et al., the similarity of *A. altibaeticus* and *A. maroccanus* suggests a common Baetic–Rifan ancestor whose divergence into two lineages must have taken place at least 5 Myr ago, when the Gibraltar Strait was opened, isolating the Iberian Peninsula and North Africa. Other species of the genus, *A. chreensis* Abrous-Kherbouche & Mauriès, 1996, *A. kabylianus* Abrous-Kherbouche & Mauriès, 1996, and *A. fodili* Abrous-Kherbouche & Mauriès, 1996, are distributed even further east in Algeria.

A similar distribution, which, however, in addition to North Africa, includes the Italian island Marettimo (Egadian archipelago off the western tip of Sicily) without occurring in the Iberian Peninsula, is the genus *Afropachyiulus* Schubart, 1960 (Julida, Julidae Leach, 1814). The genus comprises five species, of which four are known from North Africa (Morocco, Algeria, Tunisia) and one from Marettimo (Akkari and Enghoff 2008 and references therein). The Moroccan species *A. lepineyi* (Verhoeff, 1936) is known from Daia Chiker Cave in Taza (Verhoeff 1936).

Several millipede species occur on both sides of the Strait of Gibraltar, some of which have a much wider distribution. It is possible that these species have spread into the western Mediterranean naturally during the last few million years or during historical time with the help of humans. Another possible explanation is that, given how poorly studied the taxonomy of populations on both sides of the Strait of Gibraltar are, they are separate species, and this will only be clarified when molecular methods are applied. For example, *Macellolophus rubromarginatus* (Lucas, 1846) (Polydesmida, Chelodesmidae Cook, 1895) is largely distributed from southern Spain to northern Morocco and Algeria (Mauriès et al. 2006; Kime and Enghoff 2011). A second species, *M. diadema* (Gervais, 1836), from Gibraltar, is most likely a senior synonym of *M. rubromarginatus* (Lucas, 1846) (Mauriès et al. 2006). Likewise, *Oranmorpha guerinii* (Gervais, 1836) (Polydesmida, Paradoxosomatidae) is known from Spain, Algeria, and several islands in the Mediterranean Sea and the Atlantic Ocean (Kime and Enghoff 2011). Some species, like *Ommatoiulus albolineatus* (Lucas, 1845), *O. rutilans* (C.L. Koch, 1847), and *Proteroiulus hispanus* Schubart, 1959, only occur on both sides of the Gibraltar strait and, thus, probably represent the remnants of formerly continuous populations (Akkari et al. 2009).

*Macroxenus rubromarginatus* (Lucas, 1846) (Polyxenida, Polyxenidae Lucas, 1840) was described from Oran in Algeria and subsequently found twice on Malta and in southern Portugal (Kime and Enghoff 2011). *Phryssonotus platycephalus* (Lucas, 1846) (Polyxenida, Synxenidae Silvestri, 1923) shows a similar distribution (Algeria and Balearic Islands, Spain), while *Lophoproctinus inferus* (Silvestri, 1903) (Polyxenida, Lophoproctidae Silvestri, 1897) is distributed widely in North Africa and on the Canary Islands, the Balearic Islands, Corsica, and some other areas, but it is absent on the Iberian Peninsula (Kime and Enghoff 2011).

## Supplementary Material

XML Treatment for Cyphocallipus

XML Treatment for Cyphocallipus africanus

XML Treatment for Cyphocallipus excavatus

## ﻿Additional information

### Use of AI

No use of AI was reported.

### Author ORCIDs

Pavel Stoev https://orcid.org/0000-0002-5702-5677

Nesrine Akkari https://orcid.org/0000-0001-5019-4833

### Data availability

All of the data that support the findings of this study are available in the main text.
